# Methanotrophs Contribute to Nitrogen Fixation in Emergent Macrophytes

**DOI:** 10.3389/fmicb.2022.851424

**Published:** 2022-04-11

**Authors:** Jing Cui, Meng Zhang, Linxia Chen, Shaohua Zhang, Ying Luo, Weiwei Cao, Ji Zhao, Lixin Wang, Zhongjun Jia, Zhihua Bao

**Affiliations:** ^1^Ministry of Education Key Laboratory of Ecology and Resource Use of the Mongolian Plateau and Inner Mongolia Key Laboratory of Grassland Ecology, School of Ecology and Environment, Inner Mongolia University, Hohhot, China; ^2^The High School Affiliated to Minzu University of China, Hohhot, China; ^3^Inner Mongolia Key Laboratory of Environmental Pollution Control and Waste Resource Reuse, Inner Mongolia University, Hohhot, China; ^4^Institute of Soil Science, Chinese Academy of Sciences, Nanjing, China

**Keywords:** natural wetland, stable isotope analysis, nitrogen fixation, diazotrophic methanotroph, emergent plant

## Abstract

Root-associated aerobic methanotroph plays an important role in reducing methane emissions from wetlands. In this study, we examined the activity of methane-dependent nitrogen fixation and active nitrogen-fixing bacterial communities on the roots of *Typha angustifolia* and *Scirpus triqueter* using a ^15^N-N_2_ feeding experiment and a cDNA-based clone library sequence of the *nifH* gene, respectively. A ^15^N-N_2_ feeding experiment showed that the N_2_ fixation rate of *S. triqueter* (1.74 μmol h^–1^ g^–1^ dry weight) was significantly higther than that of *T. angustifolia* (0.48 μmol h^–1^ g^–1^ dry weight). The presence of CH_4_ significantly increased the incorporation of ^15^N-labeled N_2_ into the roots of both plants, and the rate of CH_4_-dependent N_2_ fixation of *S. triqueter* (5.6 μmol h^–1^ g^–1^ dry weight) was fivefold higher than that of *T. angustifolia* (0.94 μmol h^–1^ g^–1^ dry weight). The active root-associated diazotrophic communities differed between the plant species. Diazotrophic *Methylosinus* of the *Methylocystaceae* was dominant in *S. triqueter*, while *Rhizobium* of the *Rhizobiaceae* was dominant in *T. angustifolia*. However, there were no significant differences in the copy numbers of *nifH* between plant species. These results suggest that N_2_ fixation was enhanced by the oxidation of CH_4_ in the roots of macrophytes grown in natural wetlands and that root-associated *Methylocystacea*, including *Methylosinus*, contribute to CH_4_ oxidation-dependent N_2_ fixation.

## Introduction

Methane (CH_4_) is an important greenhouse gas, and natural wetlands and paddy fields are major sources of CH_4_ emissions that contribute to the global CH_4_ budget (Conrad, 2009). The Intergovernmental Panel on Climate Change (IPCC, 2007) reported that natural wetlands emit 100–231 Tg of methane to the atmosphere yearly, which represents 20–39% of the global CH_4_ emissions (Laanbroek, 2010). In wetlands dominated by emergent plants, more than 70% of the CH_4_ produced is transported through plants (Van der Nat and Middelburg, 1998; Laanbroek, 2010), and up to 90% is oxidized by aerobic methanotrophs from the root zone (Laanbroek, 2010). Aerobic methanotrophs in wetland ecosystems have been analyzed using culture-dependent and –independent approaches (Calhoun and King, 1998; Yun et al., 2013; Cai et al., 2016; Cui et al., 2020; Liu et al., 2020). Most aerobic methanotrophic strains oxidize CH_4_ using oxygen (O_2_), and they can fix N_2_ to ammonia (NH_4_^+^; Auman et al., 2001). Recently, diazotrophic methanotrophs have been reported in N-deficient environments, such as peatlands (Larmola et al., 2014; Reumer et al., 2018), forest soil (Buckley et al., 2008; Mäkipää et al., 2018), and rice paddies (Bao et al., 2014a; Shinoda et al., 2019). For example, Larmola et al. (2014) reported that *Sphagnum*-associated methanotrophs contribute to N_2_ fixation in peatland ecosystems. In addition, N_2_ fixation by *Methylosinus* (Type II methanotrophs) has been detected in rice roots without fertilization using metaproteomic approaches (Bao et al., 2014a; Minamisawa et al., 2016). Therefore, aerobic methanotrophs are not only important to reducing CH_4_ emissions, but also play a role in sustaining soil fertility (Shinoda et al., 2019).

As an important component of natural wetlands, emergent plants have a crucial impact on greenhouse gas emissions (budget; Zhang et al., 2018; Chen et al., 2019). Emergent plants, such as *Typha angustifolia*, *Scirpus triqueter*, and *Phragmites australis*, are widely distributed throughout the world and are commonly found in eutrophic wetlands (Duan et al., 2005; Laanbroek, 2010). The roots of these plant species not only play an important role in transporting the CH_4_ that is produced from anaerobic sediment but also provide a habitat for microorganisms that include methanotrophs (Nat and Middelburg, 1998; Pietrangelo et al., 2018). Studies have shown that root-associated *Methylomonas* (Type I; *Gammaproteobacteria*) is dominant in the roots of three emergent plants (*P. australis, T. angustifolia* and *S. triqueter*) using *in situ* hybridization or next-generation sequencing amplicons of *pmo*A, which encodes the particulate methane monooxgynase, based on their DNA (Fausser et al., 2012; Liu et al., 2020). However, root-associated *Methylocystis* (Type II methanotrophs, *Alphaproteobacteria*) is dominant in *S. triqueter* and *T. angustifolia* by sequencing *pmo*A gene amplicons based on cDNA (Cui et al., 2020). Owing to rapid economic development, many wetlands or lakes in the world have become eutrophic because of the excessive application of nitrogen (Bhagowati and Ahamad, 2018). Methanotrophs or diazotrophic bacteria are often affected by environmental factors, such as nitrogen, oxygen and Cu^2+^ (Hanson and Hanson, 1996; Bodelier and Laanbroek, 2004; Cheng, 2008). Whether methanotroph-mediated N_2_ fixation could occur in the roots of emergent macrophytes that grow in eutrophic natural wetlands remains unclear.

There have been methodological issues in the estimation of CH_4_ oxidation-dependent N_2_ fixation by methanotrophy (Larmola et al., 2014; Ho and Bodelier, 2015). The acetylene (C_2_H_2_) reduction assay (ARA) has been used to determine nitrogen fixation by methanotrophs (Takeda et al., 2008; Khadem et al., 2010). However, CH_4_ monooxygenase, a key enzyme for the oxidation of CH_4_ in methanotrophs, is strongly inhibited by C_2_H_2_, and CH_4_ oxidation-dependent N_2_ fixation by methanotrophs has been underestimated (Dalton and Whittenbury, 1976; Takeda, 1988). ^15^N_2_-feeding is a powerful approach to estimate the fixation of N_2_ by microorganisms (Larmola et al., 2014) and has been utilized to estimate the CH_4_ oxidation-dependent N_2_ fixation of rice plants (Shinoda et al., 2019) and the sediment of deep seas (Dekas et al., 2009).

Our goal was to demonstrate whether methanotroph-mediated nitrogen fixation occurs in the roots of *S. triqueter and T. angustifolia* in natural wetlands. This study used (i) ^15^N-N_2_ feeding experiments with and without CH_4_ to analyze the CH_4_ oxidation-dependent nitrogen fixation of *S. triqueter and T. angustifolia*, (ii) cDNA-based *nifH* gene sequencing to analyze the active root-associated nitrogen-fixing bacterial community and abundance of diazotrophic methanotrophs, and (iii) quantitative PCR of *nifH* to analyze the abundance of gene expression in the roots of the two emergent plants grown in a natural wetland.

## Materials and Methods

### Sampling Sites and Plant Materials

**FIGURE 1:**
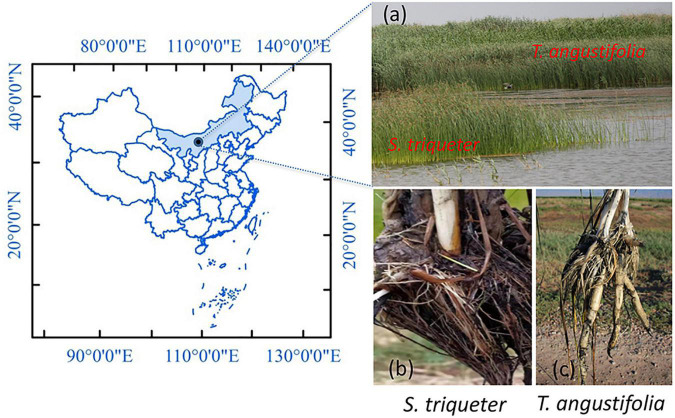
Sampling site **(a)** of *Scirpus triqueter* **(b)** and *Typhus angustifolia* **(c)** in the natural wetland of Wuliangsuhai.

*T. angustifolia* and *S. triqueter* were located in shallow water (50–70 cm) and deep water (80–100 cm) areas in the Wuliangsuhai wetland (N 40°52′36″, E 108°51′16″; Figure 1) in the Inner Mongolia Autonomous Region, China, respectively, and three to four individual plants of each species were collected on July 15, 2017 (Cui et al., 2020). The physicochemical properties of the sediments are shown in Supplementary Table 1. The roots of plants were washed with sterilized water to ensure that all of the sediment was removed, and the whole plants were divided vertically into two equal parts to collect the roots. Some of the exposed roots were collected with sterilized forceps and transferred into 50 mL Falcon tubes that contained sterile pure water (Bao et al., 2014b). All the tubes with roots were quickly snap-frozen in liquid nitrogen, immediately brought back to the laboratory, and stored at –80°C for molecular analyses.

### RNA Isolation, Preparation of cDNA Libraries and Sequencing

Total RNA was extracted from approximately 0.5 g of roots using an RNAprep Pure Plant Kit (Tiangen Biotech Co., Beijing, China) according to the manufacturer’s instructions. Since this study primarily focused on the transcriptional level, DNA contamination in the extracted RNA would affect the final experimental results. Therefore, it was imperative that the RNA be examined for possible DNA contamination before reverse transcription was conducted. The extracted total RNA was used as the template, and the 16S rRNA gene primer 27F/1492R (Martin-Laurent et al., 2001) was chosen for PCR amplification. DNA from the roots served as a positive control. PCR products were detected using 1.0% agarose gel electrophoresis and NanoVue™ Plus Spectrophotometry (GE Healthcare, Chicago, IL, United States) to ensure that there was no residual microbial DNA in the total RNA. The extracted RNA was reverse transcribed using a PrimeScript RT Reagent Kit with a gDNA Eraser (TaKaRa, Kyoto, Japan). The kit removes genomic DNA before reverse transcription, and the RT primer mixture (olige dT and random 6 mers) was used. All the samples were stored at –80°C until use.

The *nifH* gene in cDNA samples from the roots of both plants was amplified with the primers PolF (TGCGAYCCSAARGCBGACTC; Poly et al., 2001) and AQER (GACGATGTAGATYTCCTG; Wartiainen et al., 2008). Amplification and purification were conducted as previously described (Cui et al., 2020). The purified DNA fragments were linked to the pEASY-T1 vector (TransGen, Beijing, China). The products of ligation were transformed into competent cells, mixed with IPTG and X-Gal, and then coated on LB plates that contained kanamycin (100 ng/mL). White colonies were selected after overnight culture. The universal primers M13F and M13R of the vector were used for PCR detection to remove the false positive clones. Approximately 120 *nifH* positive clones for both plant samples were randomly picked from each sample for sequencing (Sangon Biotec, Beijing, China) using an ABI 3730xl DNA Analyzer (Applied Biosystems, Foster City, CA, United States) to construct the libraries. Sequences with 94% identity (Liu et al., 2021) were classified into the same operational taxonomic units (OTUs) using MOTHUR software (Schloss et al., 2009). The taxonomy of OTUs was identified by comparing the representative OTU sequences to reference *nifH* sequences using the basic local alignment search tool (BLAST) within GenBank. Each representative of the OTUs was translated to its amino acid sequence using MEGA X software (Kumar et al., 2018). After the alignment of amino acid sequences with the ClustalW program (Thompson et al., 1994), a neighbor-joining phylogenetic tree was constructed using MEGA X software (Kumar et al., 2018). The bootstrap values for each branch were determined using 1,000 iterations.

### Quantification of the *nifH* Gene

The abundances of *nifH* were quantified using quantitative PCR with a CFX Connect Optical Real-Time Detection System (Bio-Rad Laboratories, Hercules, CA, United States) with the primer set PolF/AQER (Poly et al., 2001; Wartiainen et al., 2008) for the *nifH* gene based on a cDNA library from the roots of both plants. The reactions were performed in volumes of 20 μL and contained approximately 50 ng extracted cDNA, 10 μL 2 × SYBR Premix Ex Taq (TaKaRa Biotech, Dalian, China), and 500 nM of the primers PolF and AQER (Liu et al., 2021). The PCR conditions included 40 cycles of denaturation at 94°C for 10 s, annealing at 55°C for 30 s, and extension at 72°C for 30 s. Clones of the *nifH* genes from *Methylosinus trichosporium* NCIMB 11131 (U31650) were used as the standard references.

### ^15^N_2_ Feeding Experiment for the Macrophyte Roots

Three individual plants of *T. angustifolia* and *S. triqueter* were sampled. The root systems were rinsed with water until the sediment had been completely removed. The root samples were placed in a 1 L sealing bag with inflation valves (Supplementary Figure 1), and the gas phase in the assembly was replaced with an argon (Ar)-balanced mixture of 32% (v/v) ^15^N-N_2_ (99.9 atom%; Wuhan Newradar Special Gas Co., Ltd., Wuhan, China) and 5% (v/v) O_2_ with or without 5% (v/v) CH_4_. The roots were incubated in the bag assembly at 28°C for 48 h in the dark, dried at 80°C for 3–5 days, and then powdered in a blender (Shinoda et al., 2019). Root samples without ^15^N_2_ feeding served as the negative control and were dried at 80°C immediately after they were rinsed with water. The ^15^N concentration and total N content were determined using a Stable Isotope Ratio Mass Spectrometer (MAT253, Thermo Fisher Scientific, Bremen, Germany).

The nitrogen fixation rate was calculated as follows: RW × TN/100 × (^15^Nc1 – ^15^Nc2)/^15^Ng × 100/MW, where RW is the root dry weight (g); TN is the average N content of dried root (%, w/w), and MW is the average molecular weight of N_2_. ^15^Nc1 and ^15^Nc2 represent the respective initial and final ^15^N concentrations (atom% excess) in the roots, respectively. ^15^Ng is the ^15^N concentration (atom% excess) in the N_2_ gas (Shinoda et al., 2019).

### Statistical Analysis

To test for differences in the rate of nitrogen fixation by *T. angustifolia* or *S. triqueter* roots under air, ^15^N_2_, and ^15^N_2_+CH_4_ conditions, Tukey’s honestly significant difference (HSD) test was performed using R software (ver. 3.3.2^1^) with the “multcomp” package for comparisons of multiple test samples. Significance was defined as *p* < 0.05 (Shinoda et al., 2019).

### Sequence Data Accession Numbers

The sequence data for the *nifH* gene clones from the roots of *S. triqueter* and *T. angustifolia* in this study have been deposited in the NCBI database under accession numbers MZ208474∼MZ208567 and MZ208569∼MZ208680, respectively.

## Results

### ^15^N_2_ Feeding of the Root Systems of Plants

**FIGURE 2:**
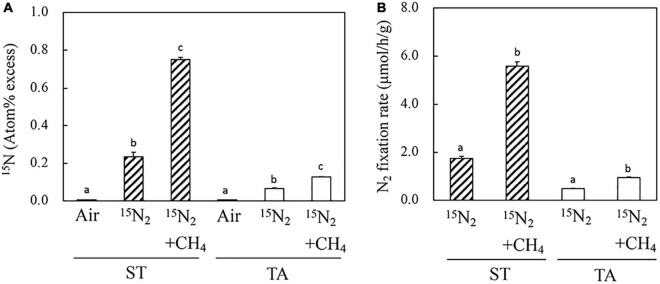
^15^N incorporation from ^15^N_2_ into roots **(A)** and the N_2_ fixation rate **(B)** of wetland-grown macrophytes in the presence and absence of 5% CH_4_. Air and ^15^N_2_, ^15^N concentrations before and after root exposure to a gas mixture of 32% (v/v) ^15^N_2_ and 5% (v/v) O_2_ in argon balance, respectively. +CH_4_, addition of 5% (v/v) CH_4_ to the gas phase for 48 h. Bars bearing the same letter (a or b) within a panel do not differ significantly according to Tukey’s test for pairwise mean comparison at α = 0.05. ST: *Scirpus triqueter*; TA: *Typha angustifolia.*

To estimate the ability of methanotrophs that inhabit the root systems of macrophytes grown in wetlands to fix nitrogen, the roots of *S. triqueter* and *T. angustifolia* were exposed to ^15^N-labeled N_2_ gas in the presence and absence of CH_4_ (Figure 2A and Supplementary Table 2). In the absence of CH_4_, the ^15^N atom percentage of *S. triqueter* roots that had been incubated was significantly higher than that of *T. angustifolia*. In contrast, the presence of CH_4_ significantly enhanced the concentration of ^15^N in the roots of both plants (Figure 2A). In particular, the ^15^N concentration of *S. triqueter* roots increased by more than threefold, which was significantly higher than that of *T. angustifolia*. The CH_4_-dependent nitrogen fixation of the *S. triqueter* roots was markedly higher than that of *T. angustifolia*. The rate of ^15^N-labeled N_2_ assimilation was calculated on the basis of the total root N content, dry weight and concentration of ^15^N (Figure 2B and Supplementary Table 2). The rate of incorporation of ^15^N-labeled N_2_ into the *S. triqueter* roots (5.58 μmol h^–1^ g^–1^ dry weight) was significantly higher than that into the *T. angustifolia* roots (0.94 μmol h^–1^ g^–1^ dry weight). This result suggests that the presence of a high concentration of CH_4_ (5%, v/v) could stimulate nitrogen fixation in the macrophyte roots, and the effect may differ among different plant species.

### Diversity of the cDNA Clone Library

A total of 240 *nifH* positive clones from both types of plant roots were sequenced (Supplementary Table 3). The library coverage was 93.6 and 86.4% in *S. triqueter* and *T. angustifolia*, respectively. *S. triqueter* had the highest number of OTUs and slightly higher diversity of all the indices compared with those of *T. angustifolia*.

### Phylogenetic Diversities of Root-Associated Diazotrophs

**FIGURE 3:**
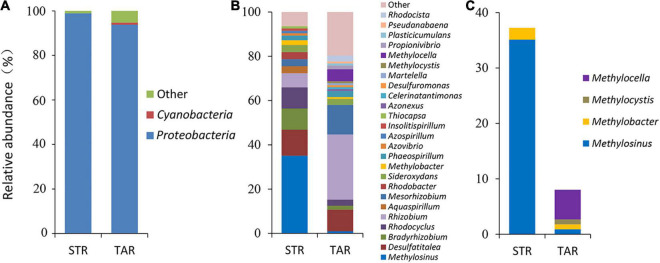
Relative abundance of active root-associated diazotrophic bacterial communities at the phylum **(A)** and genus **(B)** level and diazotrophic methanotrophic communities **(C)** in TAR and STR plants at the genus level based on a cDNA analysis of the *nifH* gene. TAR, *Typha angustifolia* roots; STR, *Scirpus triqueter* roots.

The assessment of phylogenetic compositions of diazotrophic communities revealed that *Proteobacteria* (93.8–98.9%) was dominant in both plant species at the phylum level (Figure 3A). *Rhizobiales* (52.7–54.3%) of *Alphaproteobacteria* was dominant, and *Desulfobacterales* (10.7–11.7%), *Rhodocyclales* (9.8–10.6%), *Gallionellales* (6.5–8.3%), and *Rhodospirillales* (4.3–5.4%) were minor in both libraries at the order level (Supplementary Figure 2A,B). At the family level, the diazotrophic composition clearly differed between both plant species. The relative abundances of *Methylocystaceae* (35.1%) and *Rhizobiaceae* (29.5%) indicated that they were dominant in *S. triqueter* and *T. angustifolia*, respectively. The relative abundances of *Desulfobacteraceae* (9.8–11.7%), *Rhodocyclaceae* (8.0–10.6%), *Callionellaceae* (6.3–8.5%) and *Rhodospirillaceae* (4.3–5.4%) were relatively similar in both plants, and the abundances of *Methylocystaceae* (0.9–35.1%), *Rhizobiaceae* (6.4–29.5%), *Bradyrhizobiaceae* (1.8–9.6%), and *Phyllobacteriaceae* (3.2–13.4%) clearly differed in both plants. In addition, *Beijerinckiaceae* (5.4%) and *Azonexaceae* (4.5%) were only detected in *T. angustifolia* (Supplementary Figure 2C).

Further analyses of phylogenetic compositions at lower taxonomic levels showed that *Methylosinus* (35.1%), *Desulfatitalea* (11.7%), *Bradyrhizobium* (9.6%), *Rhodocyclus* (9.6%) and *Rhizobium* (10.7%) were primarily responsible for the community shifts of Alpha/Gamma/Deltaproteobacteria in *S. triqueter* (Figure 3B). In contrast, the dominant diazotrophs (> 5%) in *T. angustifolia* were *Rhizobium* (29.5%), *Mesorhizobium* (13.4%), *Desulfatitalea* (9.8%) and *Methylocella* (5.4%), while diazotrophic *Methylocella* (5.4%) affiliated with the methanotrophs also comprised a large proportion (Figures 3B,C). The composition of root-associated diazotrophic methanotrphs clearly differed between the two plants (Figure 3C). *Methylosinus* of *Methylocystaceae* was dominant in *S. triqueter* (35.1%) and minor in *T. angustifolia* (0.9%). *Methylocella* (5.4%), *Methylobacter* (0.9%) and *Methylocystis* (0.9%) were detected from *T. angustifolia*, while only *Methylobacter* (2.1%) was detected from *S. triqueter* (Figure 3C).

**FIGURE 4:**
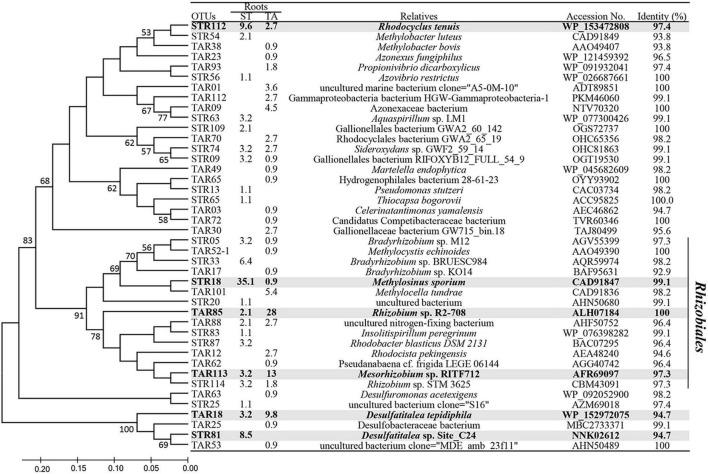
Phylogenetic distribution of representative operational taxonomc units (OTUs) of (≥ 94% amino acid identity) based on translated *nifH* gene sequences from the roots of *Scirpus triqueter* and *Typha angustifolia*. The table shows the relative abundance of OTUs in each library and the results of a BLAST search using the representative sequences. The tree was constructed using the neighbor-joining method, and the bootstrap values (%) for each branch were determined using 1,000 iterations. Bootstrap values (> 50%) are shown to the left of nodes in the tree. The main OTUs (> 5% relative abundance) are shown in gray.

Clustering analysis of the *nifH* sequences enabled the identification of OTUs responsible for the population shifts of Alpha-Beta and Deltaproteobacteria at the species level (Figure 4). The most abundant OTUs STR18 (*S. triqueter*) and TAR85 (*T. angustifolia*) exhibited 99.1% and 99.5% sequence identity to the *nifH* sequences of *Methylosinus sporium* and *Rhizobium* sp. R2-708, respectively (Figure 4). Other clones STR112, STR33, TAR85, and TAR181 were also present at much higher levels in the roots of *S. triqueter* than those of *T. angustifolia* and were identified as *Rhodocyclus tenuis* (97.4%), *Bradyrhizobium* sp. BRUESC984 (98.2%), and *Desulfatitalea* sp. Site_C24 (94.7%), respectively. In contrast, clones TAR101, TAR113, and TAR18 were detected more abundantly in the roots of *T. angustifolia* than those of *S. triqueter* and were identical to *Rhizobium* sp. R2-708 (100%), *Methylocella tundra* (98.2%), *Mesorhizobium* sp. RITF712 (97.3%), and *Desulfatitalea tepidiphila* (94.7%), respectively. In addition, a phylogenetic analysis of diazotrophic methanotrophs showed that the representative clones of five OTUs (STR18, TAR101, TAR521, STR54, and TRA38) were affiliated with known genera of methanotrophs, such as *Methylosinus*, *Methylocella*, *Methylocystis* and *Methylobacter* (Supplementary Figure 3).

### Copy Numbers of the *nifH* Gene Based on cDNA

**FIGURE 5:**
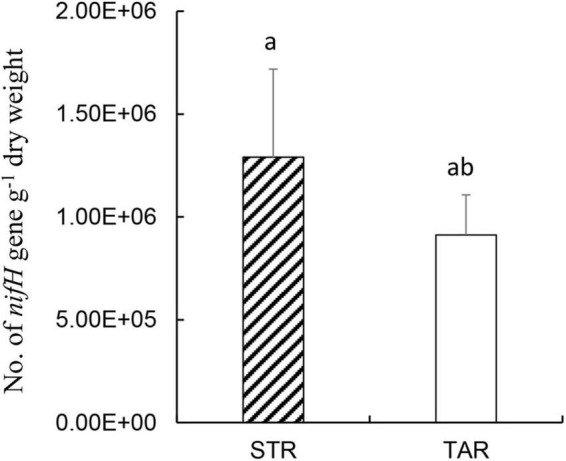
Numbers of *nifH* gene copies based on cDNA in the root samples from STR and TAR plants during July in the wetlands. Bars represent standard errors (*n* = 3). Bars with different letters (a, ab) are not significantly different (*p* < 0.05) according to Tukey’s test for pairwise mean comparison at α = 0.05. STR, *Scirpus triqueter* roots. TAR, *Typha angustifolia* roots.

To estimate the population levels of active diazotrophs, we conducted real-time quantitative PCR of the root samples of *S. triqueter* and *T. angustifolia* from the wetlands based on RNA (cDNA; Figure 5). The copy numbers of *nifH* in the roots of *S. triqueter* and *T. angustifolia* ranged from 10^5^ to 10^6^ and did not differ significantly between these two plants (Figure 4; *P* > 0.05).

## Discussion

In this study, we present direct evidence that shows that CH_4_-dependent ^15^N_2_ fixation occurred in the roots of two different emergent macrophytes (*S. triqueter* and *T. angustifolia*) grown in a natural wetland (Figure 2A). CH_4_-stimulated N_2_ fixation occurred in an N-sufficient environment, which combined two different processes of research on N_2_ fixation and methanotrophs in eutrophic wetlands.

Plant hosts have a substantial influence on the distribution of microorganisms. The bacterial communities of different plants, including roots, stems and leaves, vary between plant species (Bulgarelli et al., 2013; Pietrangelo et al., 2018). The same type of regulation was found in studies of root methanotrophs; the difference in plant species strongly affected the community structure (Yoshida et al., 2014; Liu et al., 2020) and level of expression of the methanotrophs (Cui et al., 2020). In this study, we compared the structure of diazotrophic communities in the roots of *S. triqueter* and *T. angustifolia*. Although the habitats of the two plants were similar, the activity of communities on their roots differed notably. *S. triqueter* primarily used methanotrophs for nitrogen fixation, while *T. angustifolia* depended on *Rhizobium* for nitrogen fixation. This suggests that the species of plant also affected the diazotrophic community.

Type II methanotrophs, particularly *Methylosinus*, have been proven to fix N_2_ in rice roots (Bao et al., 2014a; Shinoda et al., 2019). In this study, we found that the abundance of *Methylosinus* in *S. triqueter* was much higher than that in *T. angustifolia*, and the ability of the microorganisms on *S. triqueter* to fix N_2_ was also significantly higher according to the ^15^N_2_ feeding experiment. Therefore, the abundance of *Methylosinus* positively correlated with the rate of fixation of N_2_ on the roots. This was consistent with the research of Bao et al. (2014a) on CH_4_-dependent ^15^N_2_ fixation flora in rice. These results showed that CH_4_-dependent nitrogen fixation is common in the roots of many emergent plants, and *Methylosinus* played a significant role in CH_4_-dependent nitrogen fixation.

Nitrogen fixation, the process by which N_2_ is converted into ammonia (NH_3_) via the enzyme nitrogenase, is often inhibited by O_2_ (Cheng, 2008; Reed et al., 2011). Compared with *T. angustifolia*, *S. triqueter* was primarily distributed in shallow water areas in wetlands in this study. Thus, the high concentration of oxygen in the root system favors the habitat of rhizospheric diazotrophic methane-oxidizing bacteria. Moreover, the ability of *Methylosinus* to fix nitrogen was resistant to high concentrations of O_2_ (Shinoda et al., 2019). In contrast, *T. angustifolia* is primarily distributed in deep waters in wetlands (Cui et al., 2020) where the root system has relatively oxic conditions because of the large amount of O_2_ diffusion (Clevering et al., 1996). This could explain why the root-associated *Methylosinus* of *S. triqueter* was found in abundance, and the plant had a greater ability to fix nitrogen compared with *T. angustifolia* (Figures 2–4). Moreover, other minor methanotrophs, such as *Methylocella*, *Methylocystis* and *Methylobacter*, may also contribute to CH_4_ oxidation-dependent nitrogen fixation in this study. Type II methanotrophs, such as *Methylosinus* and *Methylocella*, are likely to contribute to nitrogen fixation in forest soil and rice plants (Buckley et al., 2008; Bao et al., 2014a; Mäkipää et al., 2018; Shinoda et al., 2019). These results were consistent with those of this study and suggest that aerobic Type II methanotrophs are widely distributed diazotrophs in natural conditions. However, the dominant diazotrophic *Methylosinus* in this study did not clearly correspond with the root-associated *Methylocystis* (Type II methanotrophs) that were dominant in *S. triqueter* and *T. angustifolia* based on cDNA amplicons of the *pmoA* gene (Cui et al., 2020). This may be owing to the fact that the amplified sequence of *pmo*A gene (510 bp) exceeds the sequence range of the MiSeq sequencing platform (< 500 bp), resulting in inaccurate BLAST results of the sequences. Moreover, it is well known that the PCR-based amplification approach also results in bias (Suzuki and Giovannoni, 1996; Sipos et al., 2007). To elucidate this methodological issue, a metatranscriptomic and/or metaproteomic analysis of root-associated microbiome should be conducted to validate the hypothesis that *Methylosinus* simultaneously contributes to both CH_4_ oxidation and N_2_ fixation in natural wetlands.

The concentration of mineral nitrogen (NH_4_^+^ or NO_3_^–^) is also one of the main factors that affects nitrogen fixation (Reed et al., 2011). Previous studies on nitrogen fixation by methane-oxidizing bacteria have primarily focused on nitrogen-deficient environments (Bao et al., 2014a,b; Larmola et al., 2014; Shinoda et al., 2019). Interestingly, although Wuliangsuhai is a relatively eutrophic wetland (Cui et al., 2020), methane oxidation-dependent nitrogen fixation still occurred in the roots of emergent macrophytes (Figure 1). Nitrogen fixation has recently been found to occur under high nitrogen conditions in forest soils because of the high C/N ratio (Zheng et al., 2020). In wetlands, the CH_4_ produced from anaerobic sediment as carbon and energy sources aids in nitrogen fixation by root-associated methanotrophs (Bao et al., 2014a). In addition, the concentrations of NH_4_^+^ are very low in the root zone of macrophytes, such as *S. triqueter*, during the growing season (Bodelier et al., 1996). Therefore, the plants might require more nitrogen for growth, and low N availability is conducive to N_2_ fixation (Reed et al., 2011). This result suggests that the nitrogen fixation of root-associated methanotrophs depends on plant species and can occur in nitrogen-sufficient environments, such as eutrophic wetlands.

In addition to methanotrophs, other diazotrophs, including *Rhizobium, Bradyrhizobium and Mesorhizobium*, are well known to fix nitrogen and are frequently detected together with methanotrophs from rice (Bao et al., 2014a; Ikeda et al., 2014; Liu et al., 2021) and other plants (Hara et al., 2019; Yoneyama et al., 2019). These nitrogen-fixing bacteria are methylotrophs and can utilize the methanol produced from plants (Hiroyuki et al., 2015; Macey et al., 2020) or from the methane oxidation process by methanotrophs (Hanson and Hanson, 1996). In addition, methylotrophs are dominant in land plant-associated soil (Macey et al., 2020), and over 40% of the diazotrophs are methylotrophs in rice roots (Liu et al., 2021). Methanotrophs typically produce methanol via the first step of methane oxidation process, and diazotrophic methylotrophs may utilize the methanol as carbon and energy sources to fix N_2_ (Bao et al., 2014a; Liu et al., 2021). Therefore, root-associated methanol-utilizing nitrogen-fixing bacteria cannot be ignored in wetlands. These results suggest that C1-cycling bacteria, including methanotrophs and methylotrophs, in the root zones of aquatic plants are important for the reduction of greenhouse gas methane and increase the benefits for stimulation of plant growth by biofertilizers.

In summary, this study revealed that CH_4_ oxidation-dependent nitrogen fixation clearly occurred in the root tissues of emergent plants (*S. triqueter* and *T. angustifolia*) in natural wetlands, and it differed between plant species. A cDNA-based *nifH* gene sequencing analysis suggests that root-associated aerobic methanotrophs, in particular *Methylosinus* (type II methanotroph), contributed to CH_4_ oxidation-dependent N_2_ fixation. In addition, the plant species had a significant effect on the root-associated diazotrophic communities. Following our previous studies on the CH_4_ oxidation-dependent N_2_ fixation of rice fields, this study provides evidence that aerobic methanotrophs fix N_2_ in the roots of emergent plants, and they have a potential role in the reduction of CH_4_ emissions and enhancement of plant growth in natural wetlands.

## Data Availability Statement

The datasets presented in this study can be found in online repositories. The names of the repository/repositories and accession number(s) can be found in the article/Supplementary Material.

## Author Contributions

ZB and JZ designed the study. ZB, JC, MZ, LC, and SZ performed the experiments. JC, YL, WC, LW, ZJ, JZ, and ZB analyzed the data. ZB and JC wrote the manuscript. All authors contributed to the article and approved the submitted version.

## Conflict of Interest

The authors declare that the research was conducted in the absence of any commercial or financial relationships that could be construed as a potential conflict of interest.

## Publisher’s Note

All claims expressed in this article are solely those of the authors and do not necessarily represent those of their affiliated organizations, or those of the publisher, the editors and the reviewers. Any product that may be evaluated in this article, or claim that may be made by its manufacturer, is not guaranteed or endorsed by the publisher.

## Footnotes

1
https://www.r-project.org/
