# Supplementary figures and images for: Methanotrophs Contribute to Nitrogen Fixation in Emergent Macrophytes
Source: Front Microbiol. 2022 Apr 11;13:851424. doi: 10.3389/fmicb.2022.851424 (PMC9036440; doi:10.3389/fmicb.2022.851424)

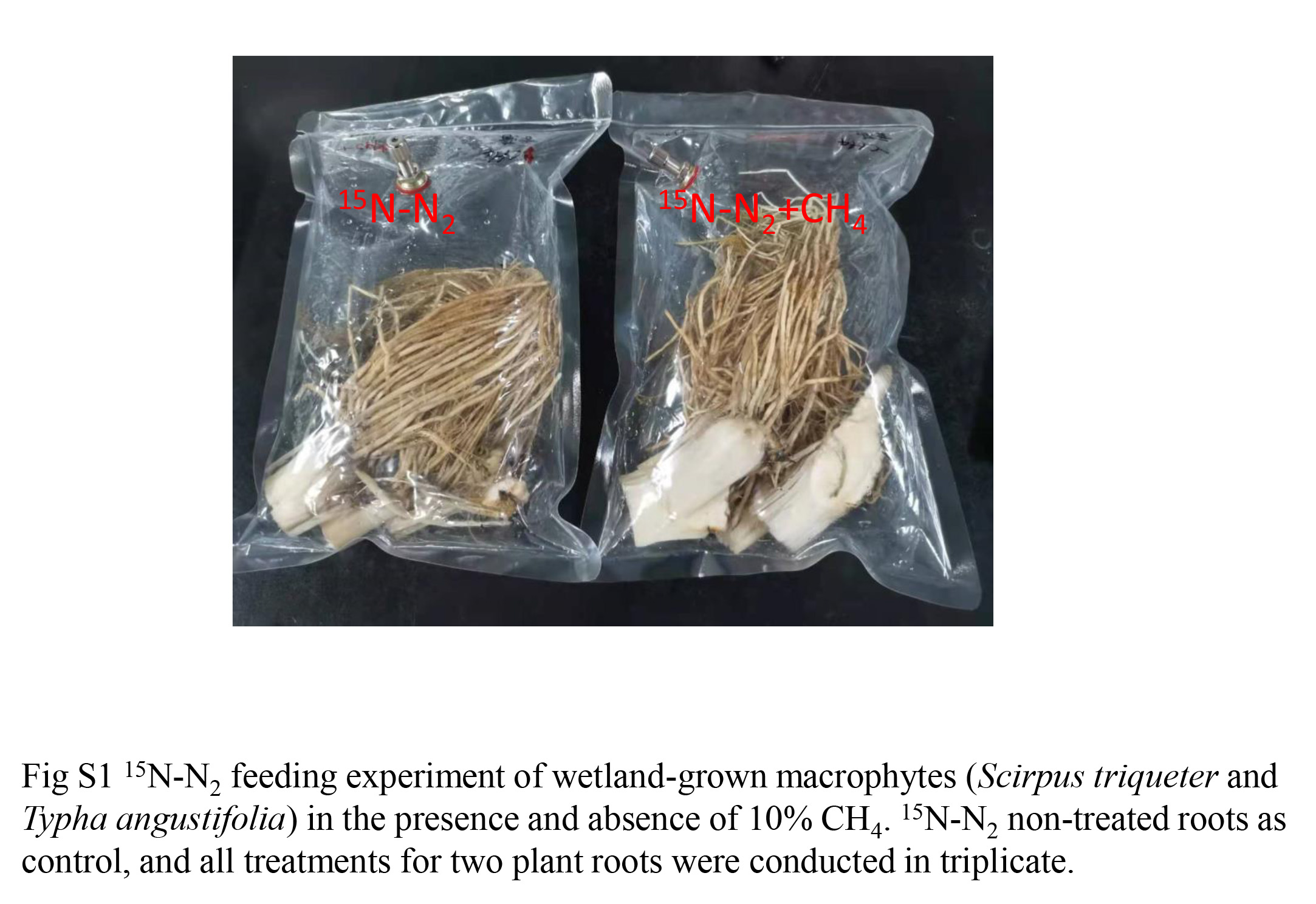

Supplement: Supplementary file 1 [file Image_1.JPEG]

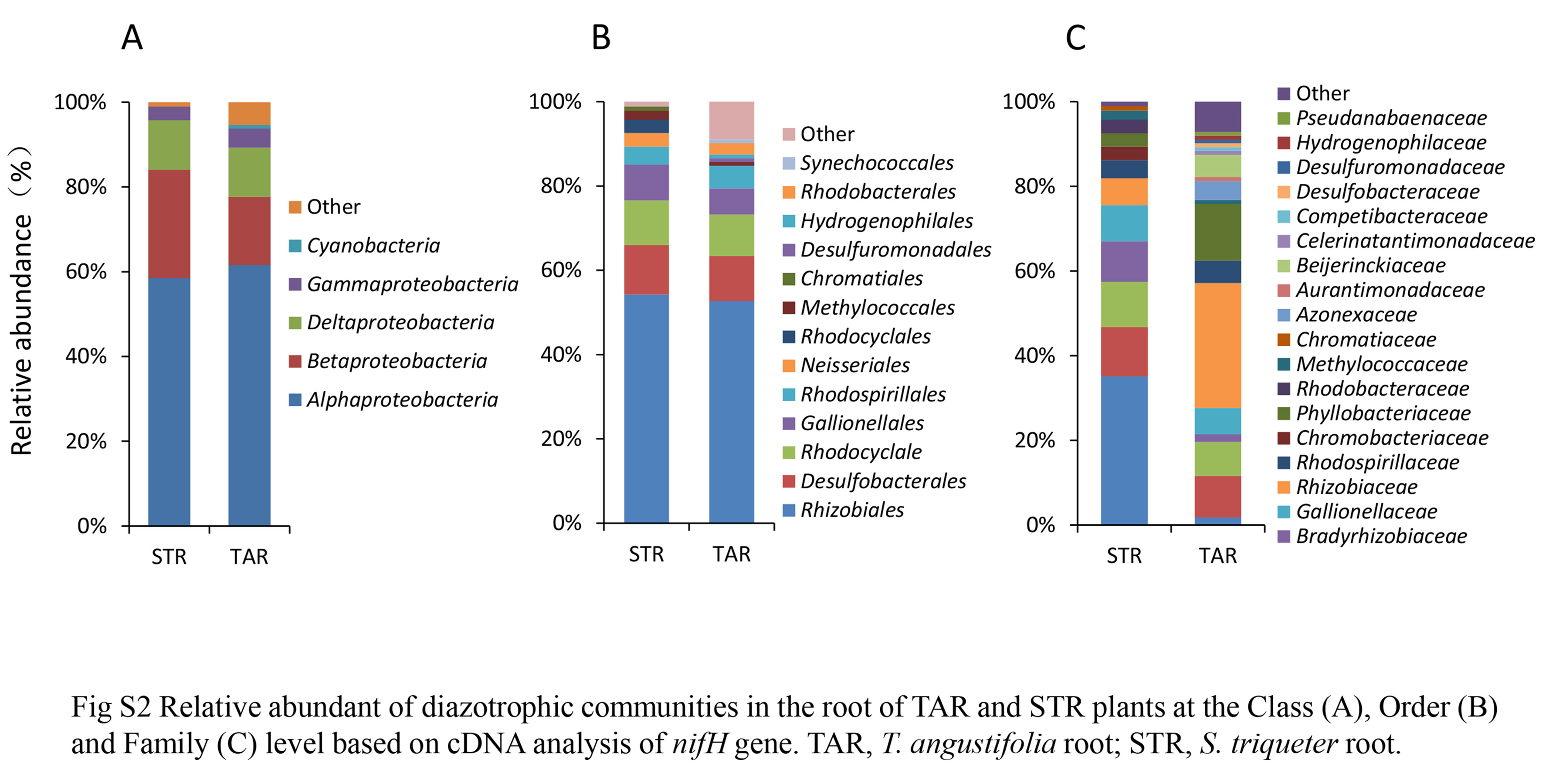

Supplement: Supplementary file 2 [file Image_2.JPEG]

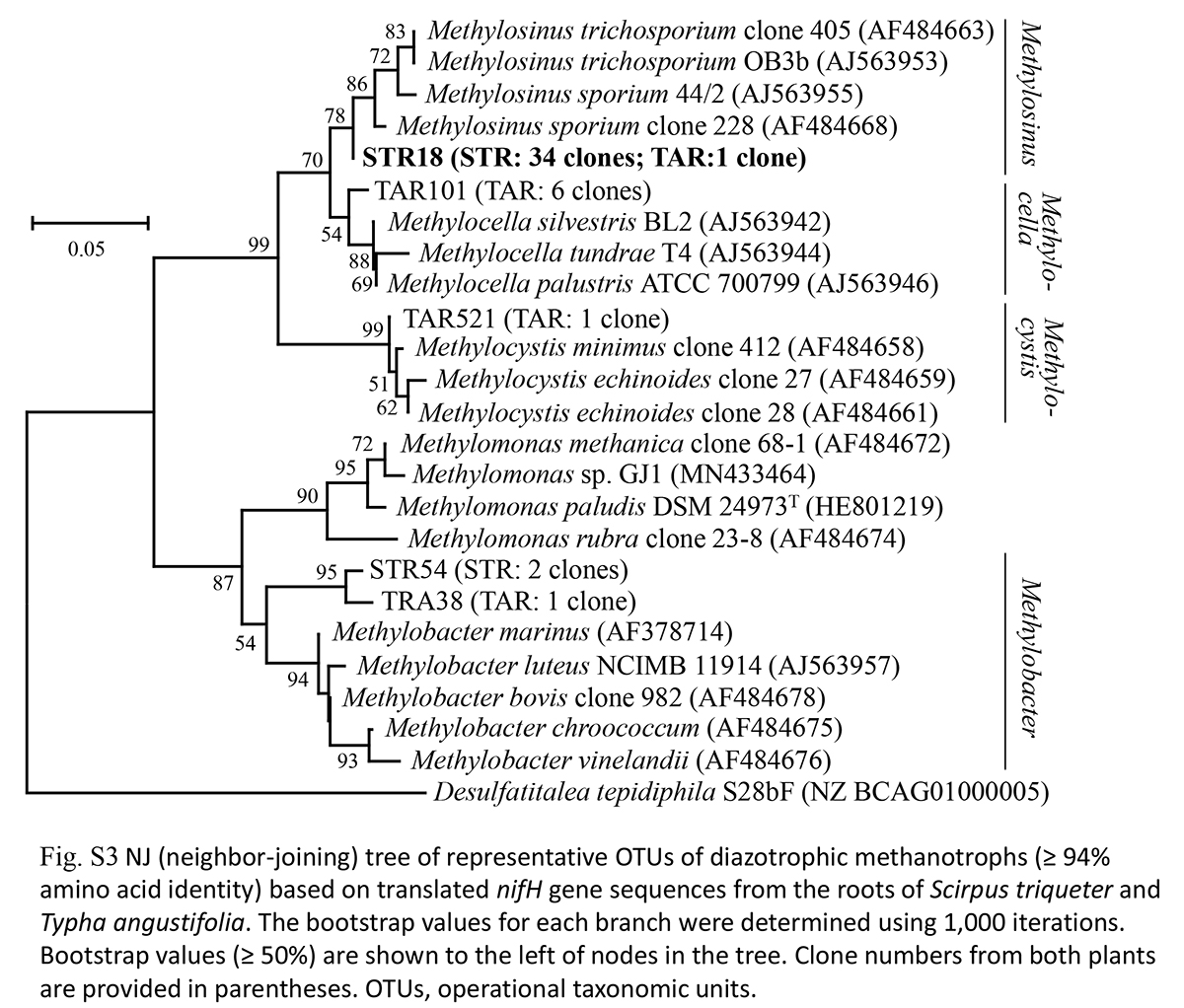

Supplement: Supplementary file 3 [file Image_3.JPEG]

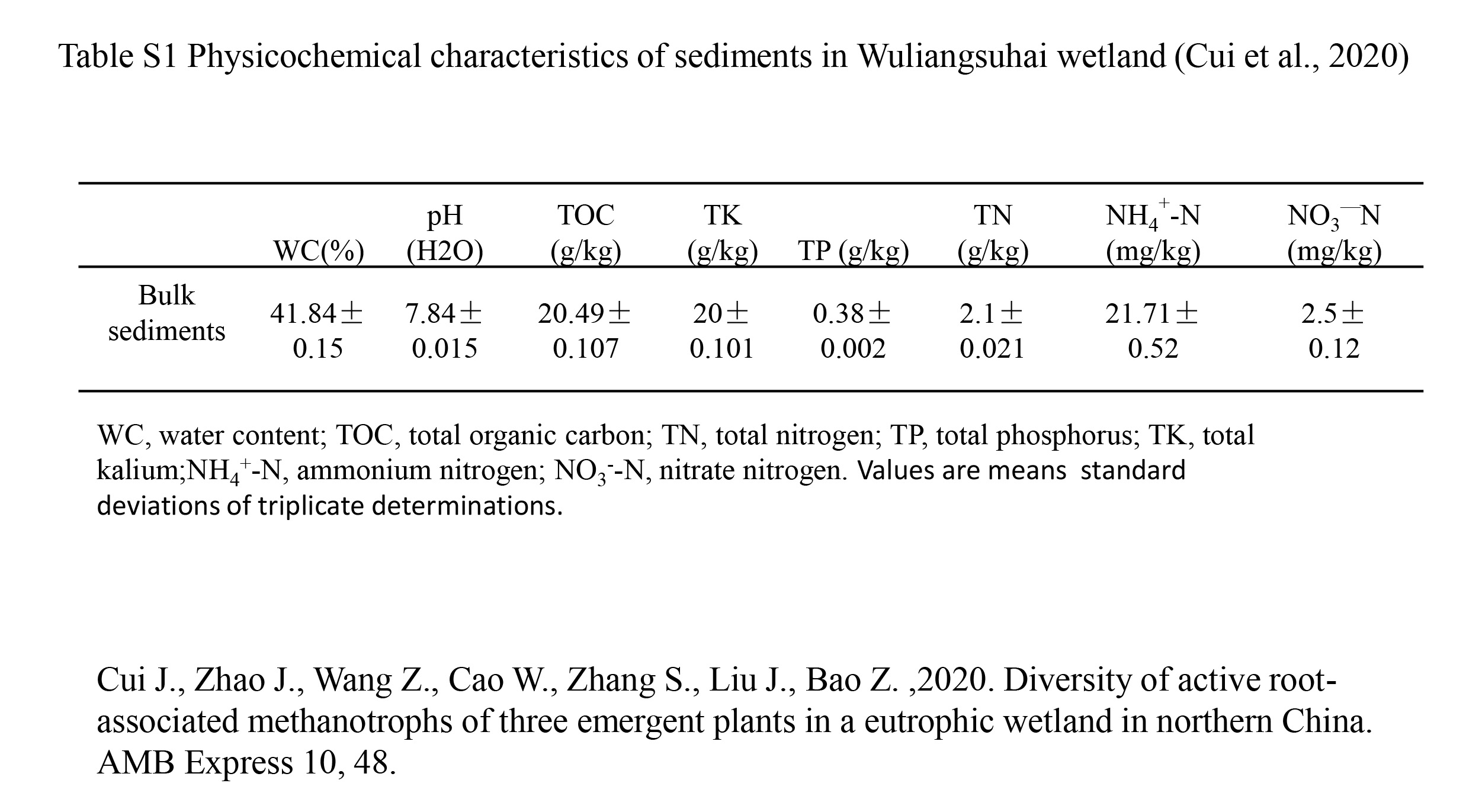

Supplement: Supplementary file 4 [file Image_4.JPEG]

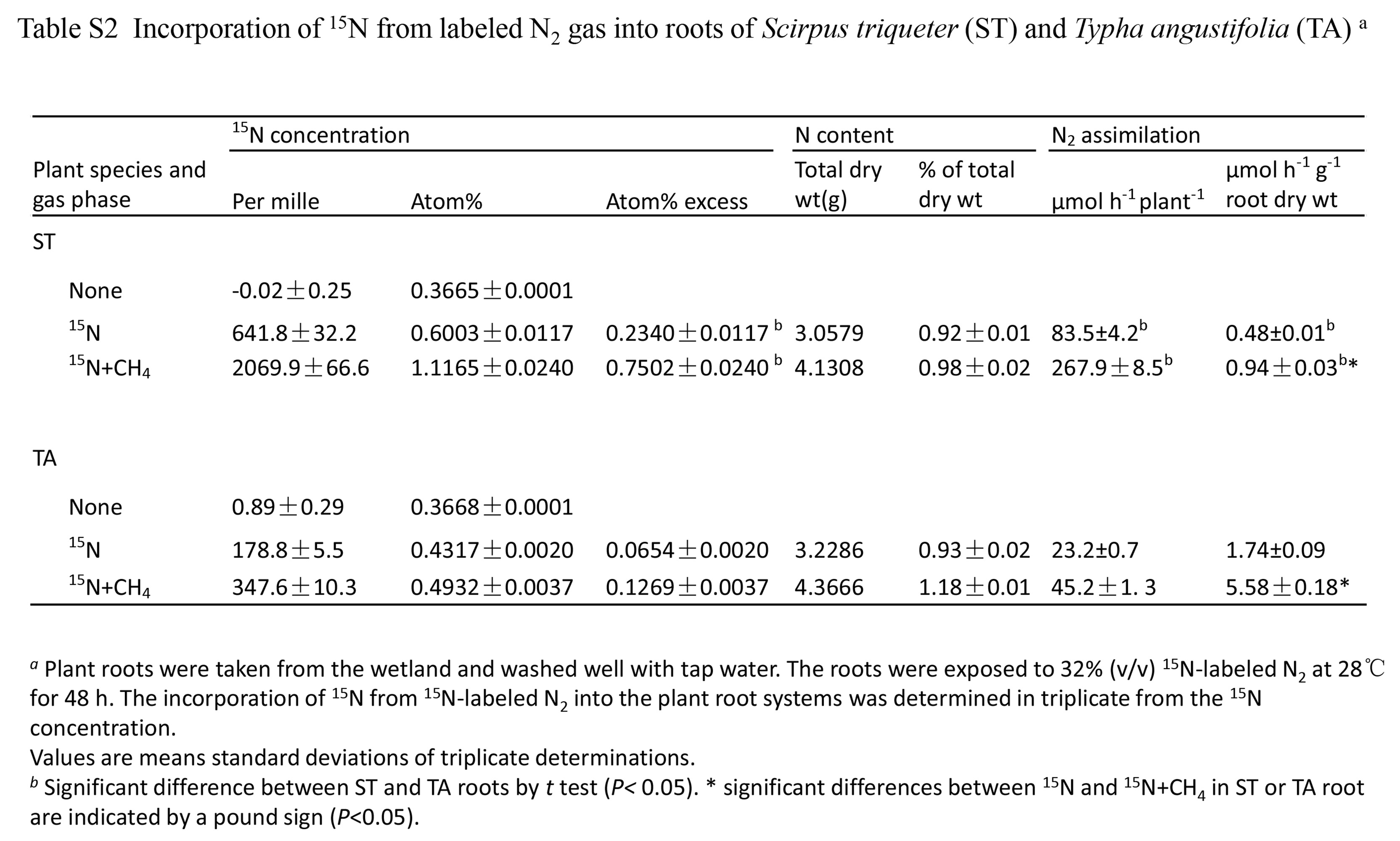

Supplement: Supplementary file 5 [file Image_5.JPEG]

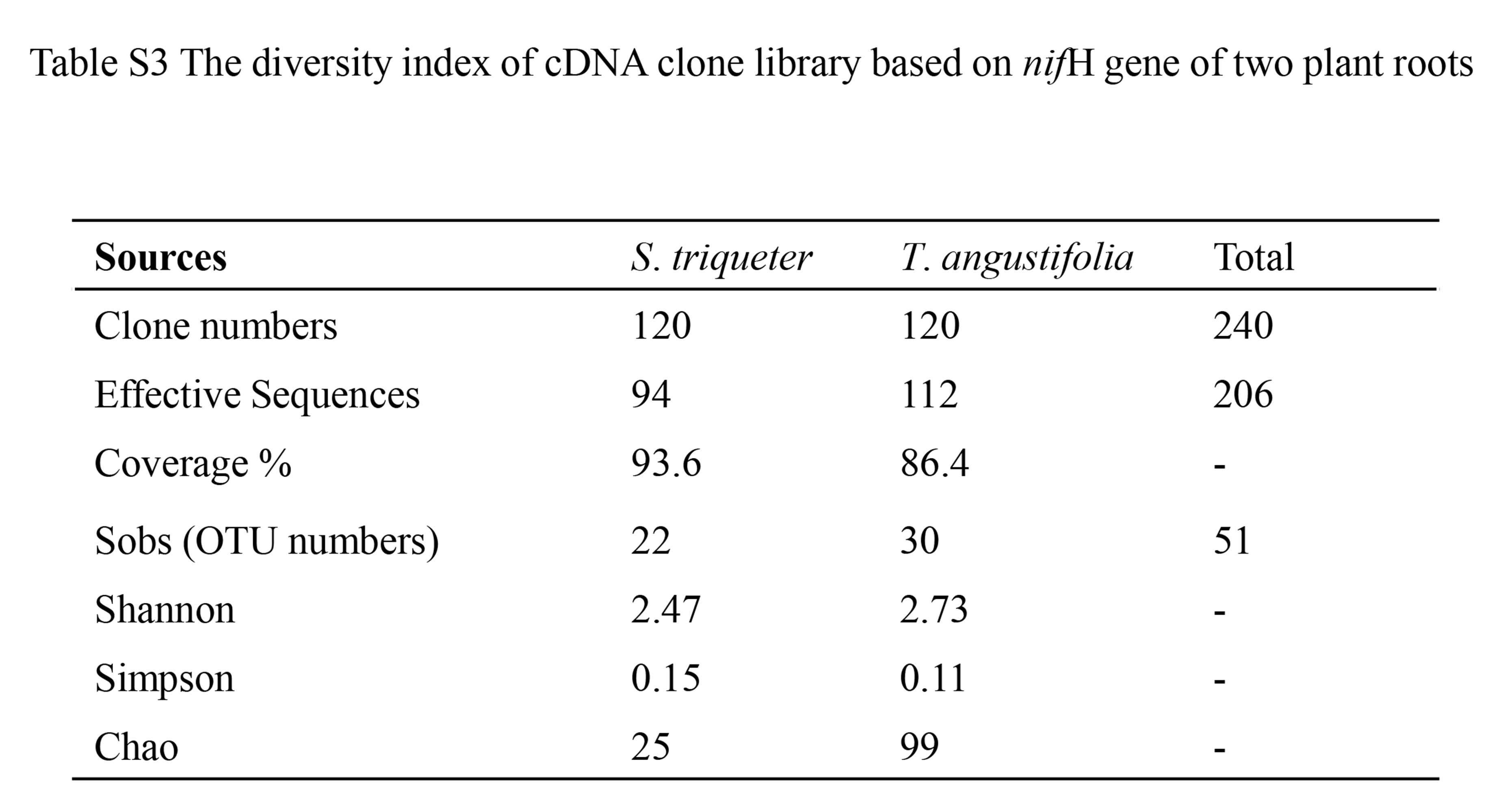

Supplement: Supplementary file 6 [file Image_6.JPEG]
